# Alterations of androgen receptor-regulated enhancer RNAs (eRNAs) contribute to enzalutamide resistance in castration-resistant prostate cancer

**DOI:** 10.18632/oncotarget.9535

**Published:** 2016-05-21

**Authors:** Jingwen Zhao, Yu Zhao, Liguo Wang, Jun Zhang, R. Jeffrey Karnes, Manish Kohli, Guixia Wang, Haojie Huang

**Affiliations:** ^1^ Department of Endocrinology and Metabolism, The First Hospital of Jilin University, Changchun, Jilin 130021, China; ^2^ Department of Biochemistry and Molecular Biology, Mayo Clinic College of Medicine, Rochester, MN 55905, USA; ^3^ Division of Biomedical Statistics and Informatics, Mayo Clinic College of Medicine, Rochester, MN 55905, USA; ^4^ Department of Laboratory Medicine and Pathology, Mayo Clinic College of Medicine, Rochester, MN 55905, USA; ^5^ Department of Urology, Mayo Clinic College of Medicine, Rochester, MN 55905, USA; ^6^ Department of Oncology, Mayo Clinic College of Medicine, Rochester, MN 55905, USA; ^7^ Mayo Clinic Cancer Center, Mayo Clinic College of Medicine, Rochester, MN 55905, USA

**Keywords:** androgen receptor, castration resistant prostate cancer (CRPC), enzalutamide, therapy resistance, eRNA

## Abstract

Enzalutamide is a second-generation anti-androgen for treatment of castration-resistant prostate cancer (CPRC). It prolongs survival of CRPC patients, but its overall survival benefit is relatively modest (4.8 months) and by 24 months most patients progress on enzalutamide. To date, however, the molecular mechanisms underlying enzalutamide resistance remain elusive. Herein, we report enzalutamide treatment-induced alterations of androgen receptor (AR)-regulated enhancer RNAs (AR-eRNAs) and their roles in enzalutamide-resistant growth and survival of CRPC cells. AR chromatin immunoprecipitation and high throughput sequencing (ChIP-seq) and RNA-seq analyses revealed that 188 and 227 AR-eRNAs were differentially expressed in enzalutamide-resistant LNCaP and C4-2 cells, respectively. The AR-eRNAs upregulated in C4-2 cells and downregulated in LNCaP cells were selected through meta-analysis. Expression of AR-eRNAs and related mRNAs in the loci of *FTO*, *LUZP2*, *MARC1* and *NCAM2* were further verified by real-time RT-PCR. Silencing of *LUZP2* inhibited, but silencing of *MARC1* increased the growth of enzalutamide-resistant C4-2 cells. Intriguingly, meta-analysis showed that expression of *LUZP2* mRNA increased in primary tumors compared to normal prostate tissues, but decreased again in metastatic CRPC. Our findings suggest that eRNA alteration profiling is a viable new approach to identify functional gene loci that may not only contribute to enzalutamide-resistant growth of CRPC, but also serve as new targets for CRPC therapy.

## INTRODUCTION

Prostate cancer (PC) remains the most commonly diagnosed cancer and is the second leading cause of cancer deaths in American men [1]. Androgen deprivation therapy (ADT) is the mainstay of treatment for advanced PC [2]. While blockage of the AR activities through ADT initially suppresses PC growth, disease eventually evolves into castration-resistant prostate cancer (CRPC). Recent studies suggest that extra-gonadal androgen, including PC cell intracrine mechanism, can still activate the AR pathway even at a very low concentration [3–5]. These seminal findings have led to the development of the second-generation hormonal therapies including enzalutamide. Enzalutamide is a competitive AR inhibitor that inhibits AR translocation to the nucleus, co-activator recruitment, AR binding to DNA and activation of AR target genes [6]. Administration of enzalutamide to CRPC patients has achieved a prolonged overall survival [7], yet a proportion of patients do not benefit from this treatment. The overall survival benefit is relatively modest (4.8 months) [7], and by 24 months most patients progress on enzalutamide [8]. Several studies suggest that this resistance is correlated with the upregulation of androgen-related biological activities. For instance, expression of genes involved in the androgen synthesis, such as *AKR1C3*, *HSD3B* and *CYP17A1*, was significantly elevated in enzalutamide-resistant PC cells [9]. Compared to the enzalutamide-sensitive cells, upregulation of these genes potentially results in the higher levels of testosterone or dihydrotestosterone (DHT), as well as the precursors of testosterone such as cholesterol, DHEA, and progesterone in enzalutamide-resistant PC cells. Moreover, it has been confirmed that expression of the full-length AR and its splice variants is one of the major factors that drive enzalutamide-resistance in the LNCaP model [10]. Thus, it is of pivotal importance to identify and validate alternative targets for the development of therapeutic modalities to overcome enzalutamide resistance in CRPC.

Mammalian genomes are populated with thousands of enhancers that orchestrate cell-type-specific gene expression programs [11–14]. Several findings have revealed a large repository of active enhancers that can be dynamically tuned to elicit alternative gene expression programs, which may underlie many sequential gene expression related to cell differentiation and disease progression [15]. eRNAs, a new class of non-coding RNAs that are transcribed from enhancers, were initially discovered to be actively engaged in promoting mRNA synthesis in neurons [16]. Recent advance has shown that eRNA plays an important role in tumorigenesis and anticancer drug resistance in several cancers including CRPC. For example, upon activation by a series of enhancers, FOXA1 simultaneously facilitates or restricts transcription of genes regulated by AR [17–19]. Moreover, it was recently found that FOXA1 is able to trigger dramatic reprogramming of the hormonal response by causing a massive switch in AR binding to a distinct cohort of pre-established enhancers, potentially resulting in a worse prognosis in certain advanced prostate tumors [20]. However, the role and the mechanism of eRNA in the development of enzalutamide resistance in CRPC are unknown. In the present study, we explored the alterations of AR-regulated eRNAs and mRNA expression of related genes in response to the treatment of enzalutamide and their contribution to enzalutamide-resistance in CRPC.

## RESULTS

### Enzalutamide treatment promotes cytotoxic effect on LNCaP cells and cytostatic effect on C4-2 cells along with morphological changes

**Figure 1:**
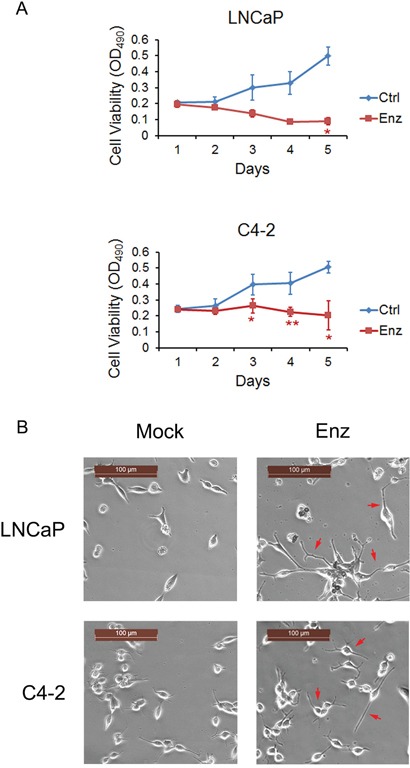
Enzalutamide treatment promotes cytotoxic effect on LNCaP cells and cytostatic effect on C4-2 cells along with morphological changes **A.** LNCaP (Upper panel) and C4-2 cells (Lower panel) were treated with 10 μM enzalutamide (Enz) for 5 days and cell viability was assessed by MTS assay. Error bars, SD from six replicates. * *P*<0.05; ** *P* < 0.01. **B.** LNCaP and C4-2 cells were treated with 10 μM enzalutamide (Enz) for 28 days and neurite outgrow (red arrows) was detected in both cell lines. Scale bars, 100 μm.

To assess the biological effect of enzalutamide on androgen sensitive and castration-resistant PC cells, LNCaP and C4-2 cells were employed as corresponding models to examine for their responses to short and long-term enzalutamide treatment. To determine the short-term responses to enzalutamide treatment, LNCaP and C4-2 cells were treated with enzalutamide (10 μM) for 5 days and cell viability was assessed by MTS assay. The number of LNCaP cells treated with DMSO (vehicle control) increased from day 1 to 5, and decreased when treated with 10 μM enzalutamide (Figure 1A, upper panel). The number of C4-2 cells treated with DMSO also increased from day 1 to 5, but remained static when treated with enzalutamide (10 μM) (Figure 1A, lower panel). These results indicate that enzalutamide was cytotoxic to LNCaP cells, but cytostatic to C4-2 CRPC cells following short-term treatment.

To determine the long-term responses to enzalutamide treatment, LNCaP and C4-2 cells were treated with 10 μM enzalutamide for 28 days and neurite outgrowth was detected (Figure 1B). These results are consistent with previous studies that neuroendocrine differentiation in PC can be induced by ADT or inhibition of the AR *in vitro* [21–26], in PC xenografts in mice [27–31] and in patient samples [32, 33]. While a very small number of LNCaP cells survived, most of them died after 28 days of enzalutamide treatment. In contrast, C4-2 cells developed into enzalutamide-resistant cells without obvious reduction of cell numbers. Thus, LNCaP cells are sensitive, but C4-2 CRPC cells are resistant to long-term treatment with enzalutamide.

### Identification of AR-regulated enhancer RNAs (AR-eRNA) affected by long-term enzalutamide treatment in LNCaP and C4-2 Cells

**Figure 2:**
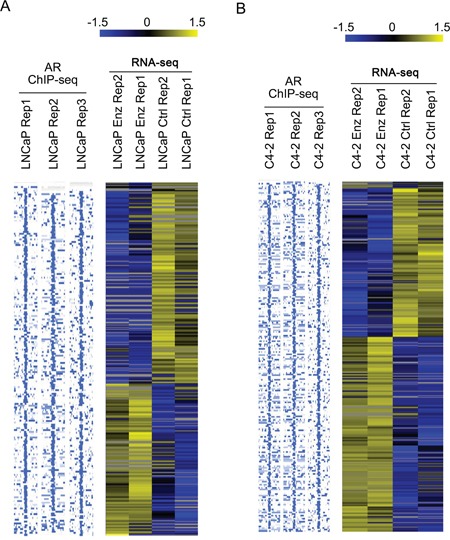
Identification of AR-regulated enhancer RNAs (AR-eRNAs) affected by long-term enzalutamide treatment in LNCaP and C4-2 cells LNCaP **A.** and C4-2 cells **B.** were treated with or without enzalutamide (10 μM) for 28 days and harvested for strand-specific RNA-seq experiments. To define the eRNAs expressed from AR binding sites, AR ChIP-seq was performed using anti-AR antibody (N20, Santa Cruz Biotechnology) in both LNCaP and C4-2 cells treated with androgen (1 nM mibolerone, a synthetic androgen). AR ChIP-seq and RNA-seq data analysis was performed to define up- and down-regulated AR-eRNAs in LNCaP and C4-2 cells treated with enzalutamide. Heat maps were used to show differentially expressed AR-eRNAs in LNCaP (A) and C4-2 (B) cells treated with or without enzalutamide. Each row on the heat map represents a probe set; each column represents an individual sample. Gene expression on each probe set was standardized to the mean of samples where red color is higher than the mean and green color is lower than the mean.

**Figure 3:**
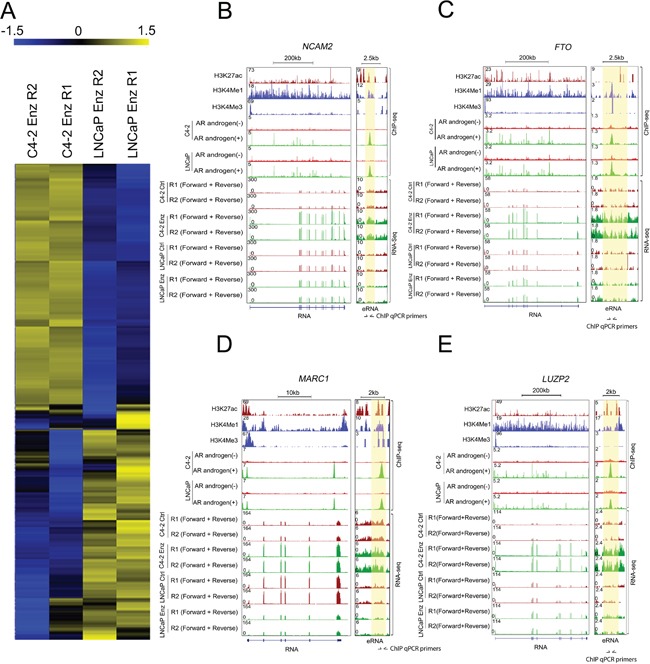
Identification of AR-eRNAs whose expression is altered in LNCaP and C4-2 cells after long-term treatment with enzalutamide **A.** Heat maps show AR-eRNAs differentially regulated in LNCaP and C4-2 cells after long-term treatment with enzalutamide. **B-E.** Screenshots of the UCSC genome browser show AR-eRNAs (right panel) and related mRNAs (left panel) expression in the loci of *NCAM2* (B) *FTO* (C) *MARC1* (D) and *LUZP2* (E) The area highlighted in orange indicates the enhancer region expressing AR-eRNA in each locus.

To identify AR-eRNAs that are possibly responsible for development of enzalutamide resistance, LNCaP and C4-2 cells that survived after 28-day treatment of enzalutamide (10 μM) were subjected to RNA-seq analyses. We also performed AR ChIP-seq in LNCaP and C4-2 cells treated with or without androgen to define the AR-regulated eRNAs. Following long-term treatment with enzalutamide, 188 and 227 AR-eRNAs were identified to be affected in LNCaP and C4-2 cells, respectively. Heat maps show differentially expressed AR-eRNAs in LNCaP (Figure 2A) and C4-2 (Figure 2B) cells after treated with enzalutamide. Furthermore, we found that 102 (54.3%) out of 188 AR ChIP-seq peaks in LNCaP cells and 151 (66.5%) out of 227 AR ChIP-seq peaks in C4-2 cells overlap with AR ChIP-exo peaks in LNCaP cells treated with DHT (P < 0.001) [34]. Given that agonist failed to induce AR binding in antagonist responsive regions and vice versa [34], the enhancers we report here should belong to the agonist responsive regions as defined by Chen et al [34]. This finding is not only consistent with our observation that AR binding at these sites was largely enhanced by androgen treatment in both LNCaP and C4-2 cells (Figure 3B–3E, see below), but also consistent with the finding of Chen and colleagues that DHT-induced AR binding at these sites was inhibited by enzalutamide treatment [34]. These findings suggest that expression of the identified AR-eRNAs can be regulated by AR and may contribute to the development of enzalutamide resistance in CRPC cells.

### Identification of AR-eRNAs and related mRNAs differentially regulated by enzalutamide in C4-2 and LNCaP cells

Since C4-2 cells were more resistant to enzalutamide compared to LNCaP cells as shown in Figure 1, we aimed to identify upregulated oncogenic genes that may contribute to enzalutamide resistance in CRPC cells. To this end, we selected AR-eRNAs upregulated in C4-2 cells while downregulated in LNCaP cells after enzalutamide long-term treatment. Heat maps show AR-eRNAs upregulated in C4-2 cells and downregulated in LNCaP cells after enzalutamide treatment and vice versa (Figure 3A). The changes are exemplified by the expression of AR-eRNAs and related mRNAs in the loci of *NCAM2, FTO*, *MARC1* and *LUZP2* (Figure 3B–3E). Enhancers at these loci are evident by enrichment of AR occupancy and enhancer histone marks H3 lysine-4 monomethylation (H3K4me1) and H3 lysine-27 acetylation (H3K27ac), but little or no enrichment of the promoter mark H3 lysine-4 trimethylation (H3K4me3) (Figure 3B–3E). RNA-seq data showed that after long-term enzalutamide treatment, the AR-eRNAs in the loci of *NCAM2, FTO*, *MARC1* and *LUZP2* increased in C4-2 cells but decreased in LNCaP cells, and accordingly, the related mRNAs increased in C4-2 but decreased in LNCaP cells, with the exceptions that the AR-eRNA in the locus of *LUZP2* and the related mRNA in the locus of *FTO* exhibited no significant change in LNCaP cells.

**Figure 4:**
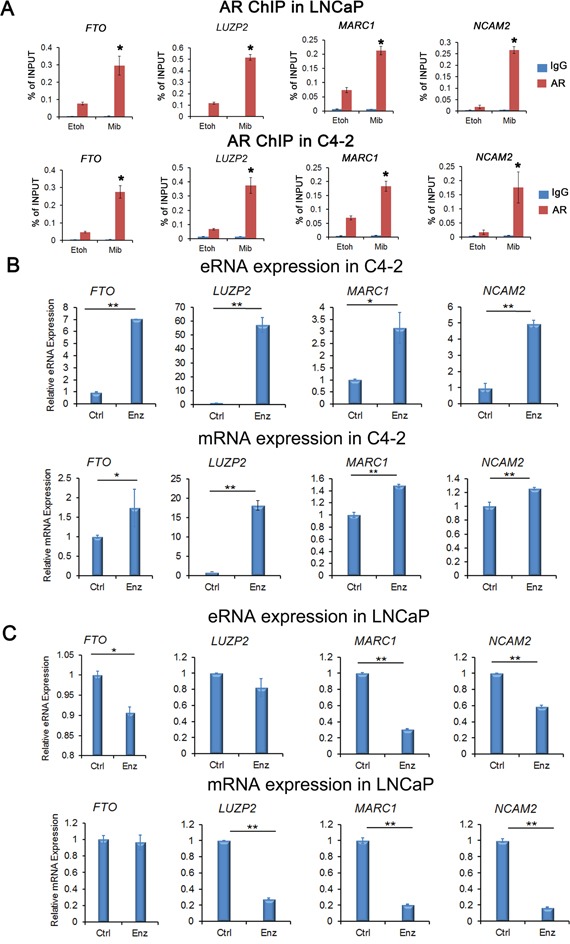
Verification of AR binding and expression of AR-eRNA and mRNA by quantitative PCR **A.** ChIP-qPCR analysis of AR binding to enhancers in the loci of *FTO*, *LUZP2*, *MARC1* and *NCAM2* in both LNCaP and C4-2 cells treated with or without mibolerone (1 nM). Immunoprecipitated DNA was detected by real-time PCR. Primer locations in the enhancer regions of these loci are shown in Figure 3B-E. All data shown are mean values ± SD (error bar) from three replicates. * *P* < 0.01. **B.** Real-time RT-PCR validation of upregulation of AR-eRNAs and mRNA of indicated genes in C4-2 cells treated with enzalutamide (10 μM) for 28 days. **C.** Real-time RT-PCR validation of downregulation of AR-eRNAs (except *LUZP2* eRNA) and mRNA (except *FTO*) of indicated genes in LNCaP cells treated with enzalutamide (10 μM) for 28 days. Columns, mean values among three replicates; error bars, SD. * *P*<0.05; ** *P* < 0.01.

ChIP-qPCR assays showed that AR was readily recruited into the enhancers of these loci (primer locations in the enhancer regions at these loci are shown in Figure 3B–3E) in both LNCaP and C4-2 cells without androgen stimulation (Figure 4A). AR recruitments at these enhancers were further enhanced by treatment of cells with mibolerone, a synthetic androgen (Figure 4A). These data verify AR recruitment to the regulatory regions of the AR-eRNAs we examined.

We also performed real-time RT-PCR to confirm the differential regulation of these genes by enzalutamide in LNCaP and C4-2 cells. We treated cells with or without enzalutamide (10 μM) for 28 days. We found that expression of AR-eRNAs and the corresponding mRNAs in the loci of *FTO*, *LUZP2*, *MARC1* and *NCAM2* were upregulated in C4-2 cells, but downregulated in LNCaP cells, except that the AR-eRNA expression in the locus of *LUZP2* and the mRNA expression in the locus of *FTO* remained almost unchanged in LNCaP cells (Figure 4B, 4C). Thus, our data confirm the regulation of four candidate gene loci *FTO*, *LUZP2*, *MARC1* and *NCAM2* by enzalutamide, suggesting that their expression may contribute to the development of enzalutamide resistance in CRPC cells.

### The enhancer-promoter interaction is enhanced in C4-2 cells after enzalutamide treatment in the loci of *NCAM2* and *MARC1*

**Figure 5:**
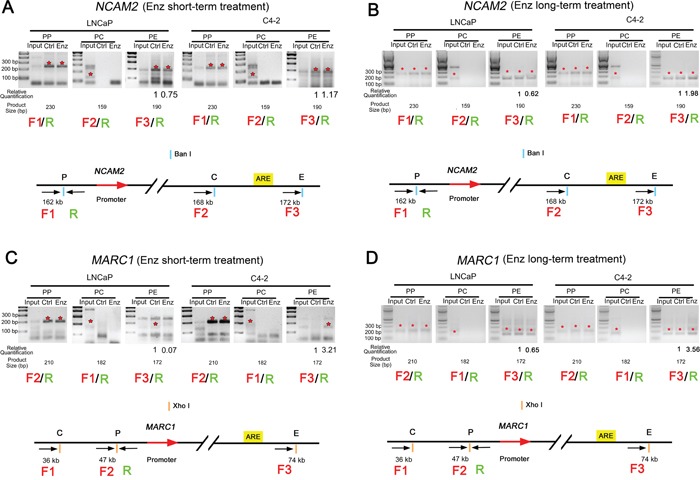
Enzalutamide treatment enhances enhancer-promoter interaction in the loci of *NCAM2* and *MARC1* in C4-2 cells The enhancer-promoter looping in the indicated gene loci was measured by 3C assay. The expected PCR amplicons of DNA obtained from 3C assay at the loci of *NCAM2* and *MARC1* are 190 and 172 bp, respectively. **A** and **B.** 3C assays in the *NCAM2* locus in both LNCaP and C4-2 cells treated with DMSO or enzalutamide (10 μM) for 48 h (A) or 28 days (B). **C** and **D.** 3C assays in the *MARC1* locus in LNCaP and C4-2 cells treated with DMSO or enzalutamide (10 μM) for 48 h (C) or 28 days (D). PP: crosslinking at promoter alone; PC: crosslinking between promoter and control (middle) sites; PE: crosslinking between promoter and enhancer. LNCaP Ctrl: LNCaP cells treated with DMSO; LNCaP Enz: LNCaP cells treated with enzalutamide; C4-2 Ctrl: C4-2 cells treated with DMSO; C4-2 Enz: C4-2 cells treated with enzalutamide. Asterisks in red: PCR products are at the expected size.

Next we performed chromatin conformation capture (3C) assays to verify that the eRNA-producing regions are truly the enhancers of putative target genes examined and to assess the impact of enzalutamide treatment on the interaction between the enhancers and promoters. C4-2 and LNCaP cells were treated with or without enzalutamide (10 μM) for 48 h and 28 days to determine the short- and long-term effect of enzalutamide on cell growth, respectively. Since both eRNA and mRNA of *MARC1* and *NCAM2* are consistently upregulated in C4-2, but downregulated in LNCaP cells after enzalutamide treatment (Figure 4), we performed 3C assay by focusing on *MARC1* and *NCAM2* loci. As expected, the PCR amplicons from the 3C assay for the crosslinking between the enhancer and promoter in the loci of *NCAM2* and *MARC1* were 190 and 172 bp, respectively. In the locus of *NCAM2*, after both short- and long-term enzalutamide treatments, the enhancer-promoter looping invariably decreased in LNCaP cells, but increased in C4-2 cells compared to the control groups (Figure 5A, 5B). In the locus of *MARC1*, weak enhancer-promoter looping was detected in LNCaP cells treated with DMSO but no obvious looping in either LNCaP cells treated with enzalutamide or in C4-2 cells treated with DMSO. In contrast, enhancer-promoter looping was readily detected in C4-2 cells after both short- and long-term treatment with enzalutamide (Figure 5C, 5D). These results indicate that in the locus of *NCAM2*, the interaction between enhancer and promoter decreased in LNCaP and increased in C4-2 cells after enzalutamide treatment, and in the locus of *MARC1* the interaction could only be readily observed in C4-2 cells treated with enzalutamide. These results suggest that *NCAM2* and *MARC1* mRNAs are truly the targets of their corresponding eRNAs and their expression in response to enzalutamide may contribute to the development of enzalutamide resistance in C4-2 cells.

### *LUZP2* knockdown suppresses and *MARC1* knockdown promotes the growth of enzalutamide-resistant C4-2 cells

**Figure 6:**
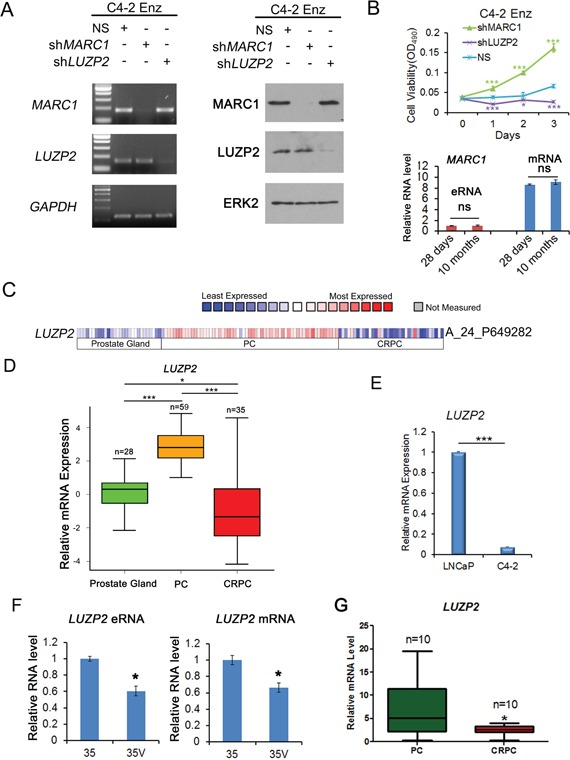
*LUZP2* knockdown suppresses and *MARC1* knockdown enhances growth of enzalutamide-resistant C4-2 cells **A.** Assessment of the effect of shRNA-mediated knockdown of MARC1 and LUZP2 by RT-qPCR and western blot analysis. Enzalutamide long-term treated C4-2 cells were treated with shRNAs for MARC1, LUZP2 or non-specific (NS) control shRNAs. Expression of MARC1, LUZP2 and GAPDH (control for off-target effect) was examined by RT-qPCR (left) and western blot (right) analysis. ERK2 was used as a protein loading control. **B.** Top panel, enzalutamide (10 μM) long-term (10 months) treated C4-2 cells were infected with non-specific (NS) control or MARC1 or LUZP2-specific shRNAs for 2 days and cell viability was measured using MTS assay for 3 additional days. Error bars, SD from six replicates. * *P*<0.05; *** *P* < 0.01. Bottom panel, RT-qPCR analysis of expression of *MARC1* eRNA and mRNA in C4-2 cells treated with enzalutamide for 28 days and 10 months. ns: no significant difference. **C.** Heat map showing expression of *LUZP2* mRNAs in a cohort of human normal prostate tissues, hormone-naïve primary prostate cancer (PC) and metastatic CRPC tissues reported by Grasso et al [35]. **D.** Box plots of *LUZP2* mRNA expression in human normal prostate tissues, primary hormone-naïve PC and CRPC tissues reported by Grasso et al [35]. Outliers were omitted from box plots. * *P* < 0.05; *** *P* < 0.001. **E.** Real-time RT-PCR analysis of *LUZP2* mRNA expression in LNCaP and C4-2 cells. Columns, mean values among three replicates; error bars, SD. *** *P* < 0.001. **F** and **G.** RT-qPCR analysis of expression of *LUZP2* eRNA and mRNA in hormone-naïve (35) and castration-resistant (35V) PDXs (F) and expression of *LUZP2* mRNA in human primary PC and CRPC tissues obtained from Mayo Clinic (G). Outliers were omitted from box plots. Columns, mean values among three replicates; error bars, SD. * *P* < 0.05.

To investigate the biological effect of knockdown of selected genes on the viability of enzalutamide-resistant CRPC cells, C4-2 cells survived after 10-month treatment with enzalutamide (10 μM) were examined for their response after treatment with shRNAs for *MARC1*, *LUZP2*, *FTO*, *NCAM2* or non-specific (NS) shRNA in the presence of enzalutamide. Since knockdown of *FTO* and *NCAM2* showed no significant effect on the viability of enzalutamide-resistant CRPC cells, we focused on *MARC1* and *LUZP2* in further studies. The knockdown effect of RNA interference in the loci of *MARC1* and *LUZP2* were confirmed by both RT-qPCR and western blot analysis (Figure 6A). The cell viability was assessed using MTS assays in cells treated with enzalutamide. We found that silencing of *LUZP2* exerted a suppressive effect on enzalutamide-resistant growth of C4-2 CRPC cells. On the contrary, silencing of *MARC1* significantly increased cell growth (Figure 6B). RT-qPCR analysis showed that there was no significant change in *MARC1* eRNA and mRNA levels after C4-2 cells were treated with enzalutamide for 28 days and 10 months (Figure 6B). These results indicate that MARC1 exerts suppressive, but LUZP2 exerts promoting effect on the growth of enzalutamide-resistant CRPC cells in culture.

As *LUZP2* plays a critical role in enzalutamide-resistant CRPC cells, we further examined its expression in normal prostate tissues, primary PC and CRPC unexposed to enzalutamide. Our analysis of a previously reported dataset [35] indicated that expression of *LUZP2* mRNA was significantly higher in primary tumors compared to normal prostate tissues, but intriguingly was lower again in metastatic CRPC compared to primary PC (Figure 6C and 6D). In line with these findings, RT-qPCR analysis showed that *LUZP2* mRNA expression was higher in hormone-naïve LNCaP cells than in C4-2 CRPC cells (Figure 6E), which is consistent with RNA-seq data in cells without enzalutamide treatment (Figure 3E). We also examined both *LUZP2* eRNA and mRNA expression in paired 35 hormone-naïve and 35V castration-resistant PDX models and demonstrated that expression of *LUZP2* eRNA and mRNA were both downregulated in CRPC samples compared to the hormone-naive counterparts (Figure 6F). Furthermore, we compared the expression of RNAs extracted from primary PC and CRPC patient tissues. We demonstrated that *LUZP2* mRNA expression was significantly higher in hormone-naïve PC patients compared with their CRPC counterparts (Figure 6G). Therefore, our data consistently showed that expression of *LUZP2* mRNA was much higher in hormone-naïve PC cells in culture, PDX models in mice and patient tissues in comparison to the CRPC counterparts, suggesting that downregulation of *LUZP2* might be a contributing factor in the development process from PC to CRPC, but its upregulation may be important for development of enzalutamide resistance.

## DISCUSSION

ADT remains the standard care for advanced PC, resulting in remission of the disease in approximately 90% of patients [36]. Unfortunately, 2–3 years after treatment, CRPC develops in most patients [37, 38]. CRPC is associated with poor prognosis [39], with the median survival time varying from 9 to 30 months and metastases in over 84% of CRPC patients which reduces the mean survival to around 14 months [40]. As a new-generation hormonal therapy, enzalutamide offers an alternative in the treatment for patients with CRPC. Unfortunately, most of them still develop resistance, stressing that further development of medical interventions of CRPC is necessary. In the realm of PC epigenomics, extensive researches have been carried out in DNA methylation, histone modifications and microRNAs [41]. The enhancer related epigenomic studies, however, remain a gap in the field of PC research. One of the most exciting discoveries in the past few years is that enhancers produce non-coding RNAs, referred to as enhancer RNAs or eRNAs [16, 20, 42]. It is generally accepted that enhancers are largely responsible for cell-type-specific gene expression [11, 14, 43]. It has been reported that DNA topoisomerase I is recruited to AR-bound functional enhancers and its activity plays an important role in regulating eRNA expression in response to DHT treatment in LNCaP cell model [44]. Hsieh et al. found that the activity of *PSA* eRNA selectively affected AR-regulated gene including *NKX3.1*, *FKBP5* and *PLZF* (also known as *ZBTB16*) [45]. However, the role of the affected eRNAs in development of resistance to the second line hormonal therapy such as enzalutamide remains to be determined. In our study, we first employed a systems biology approach to profile the differential expression of functional eRNAs at AR binding enhancers in enzalutamide-sensitive LNCaP and enzalutamide-resistant C4-2 CRPC cells. We also examined their association with nearby genes by performing high throughput screening, 3C assay and functional validation. Different from the studies mentioned above, the goal of our current study is to exploit eRNA alterations as a novel approach to identify new gene targets that are involved in the development of resistance in CRPC.

In this study, using genome-wide AR ChIP-seq and RNA-seq approaches we identified a subset of AR-eRNAs that were elevated in enzalutamide-resistant cells but reduced in sensitive cells following long-term enzalutamide treatment. The loci of *FTO*, *LUZP2*, *MARC1* and *NCAM2* have been identified and further verified to be highly altered targets that may contribute to the development of enzalutamide resistance. It has been shown recently that AR-activated enhancers marked by increased eRNAs are responsible for activation of nearby coding transcription units [20]. In two (*NCAM*2 and *MARC1*) of these 4 target genes, we have successfully confirmed the existence of the interaction between eRNA-producing enhancers and the corresponding promoters using 3C assay. The enhancer/promoter interactions in the loci of *NCAM*2 and *MARC1* in C4-2 cells were induced by enzalutamide treatment, while this interaction was suppressed by enzalutamide in the locus of *NCAM2* in LNCaP cells, suggesting that the activity of these enhancers are regulated by enzalutamide.

A significant finding from our functional studies is the potential role of *LUZP2* in development of enzalutamide resistance in C4-2 cells. *LUZP2* (leucine zipper protein 2 gene) encodes a leucine zipper protein that has been reported to be deleted in some patients with Wilms tumor-Aniridia-Genitourinary anomalies-mental Retardation (WAGR) syndrome, which is a rare congenital anomaly syndrome consisting of Wilm's tumor, aniridia, genitalanomalies and mental retardation [46, 47]. Its correlation with cancers including PCa has not yet been reported. However, our results show that *LUZP2* knockdown induces cytotoxicity in enzalutamide-resistant C4-2 CRPC cells. Our finding for the first time identifies LUZP2 as a putative therapeutic target for the treatment of enzalutamide-resistant CRPC. Moreover, our data show that *LUZP2* mRNA expression is upregulated in hormone-naïve PC compared with normal prostate tissues, but downregulated during the development from hormone-naïve PC to CRPC. However, it is upregulated again in enzalutamide-resistant CRPC compared with enzalutamide-untreated CRPC. Taken together, these results suggest an interesting gain, lose and re-gain of certain survival mechanisms during the tumorigenesis of PC, the evolution from PC to CRPC and the development of enzalutamide resistance in CRPC. Future investigation of the underlying mechanisms is warranted. Notably, it has been reported that relative to low-grade PC, high-grade cancer shows an attenuated androgen signaling signature that is similar to metastatic PC, and decreased expression of AR-dependent genes was observed during PC progression [48]. Thus, *LUZP2* gene expression pattern during PC progression resembles the phenomenon occurred in PC patients.

Another unexpected result of our study is the finding that *MARC1* knockdown promotes growth of enzalutamide-resistant CRPC C4-2 cells. The *MARC1* gene encodes signal-anchored mitochondrial protein integrated into the outer mitochondrial membrane [49]. It is expressed in liver and omental and subcutaneous fat, and pathologically participates in superoxide-mediated oxidative stress [50], but the role of MARC1 in tumorigenesis and anticancer therapy resistance remains largely unknown. Our data show that eRNA expression at this locus was elevated after long-term enzalutamide treatment, which is consistent with enzalutamide treatment-induced increase in the interaction between its enhancer and promoter in C4-2 CRPC cells. These results imply that MARC1 may function as a negative regulator that antagonizes the development of enzalutamide resistance in CRPC cells. Thus, this gene could be utilized as a potential indicator for drug sensitivity and harnessed for treatment of enzalutamide-resistant CRPC.

A limitation in our study is that we only performed in depth analysis for a few AR-eRNAs. Further studies on the regulation of expression of more AR-eRNAs in hormone-naïve PC and CRPC as well as their roles in development of enzalutamide resistance are warranted in future.

## MATERIALS AND METHODS

### Cell lines, cell culture and reagents

LNCaP cells and human embryonic kidney 293T cells were purchased from ATCC (Manassas, VA). C4-2 cells were purchased from UroCorporation. LNCaP and C4-2 cells were cultured in RPMI 1640 medium supplemented with 10% fetal bovine serum (FBS) (Thermo Fisher Scientific) and 100 μg/ml penicillin-streptomycin-glutamine (Thermo Fisher Scientific) at 37°C with 5% CO_2_. 293T cells were maintained in Dulbecco's modified Eagle's medium (Thermo Fisher Scientific) supplemented with 10% fetal bovine serum (FBS) (Invitrogen) and 100 μg/ml penicillin-streptomycin-glutamine (Thermo Fisher Scientific) at 37°C with 5% CO_2_. Enzalutamide was kindly provided by Medivation/Astellas (San Francisco, CA). Mibolerone was purchased from Steraloids Inc (Newport, RI). The antibody used for AR ChIP-seq is anti-AR (N-20) from Santa Cruz Biotechnology. The antibodies used for western blot are anti-ERK2 (D-2, Santa Cruz Biotechnology), anti-LUZP2 (ab171165, Abcam) and anti-MARC1 (D-16, Santa Cruz Biotechnology).

### Human prostate cancer specimens and RNA isolation from human tissues

Formalin-fixed paraffin-embedded (FFPE) hormone-naïve primary PC and CRPC tissues were randomly selected from the Mayo Tissue Registry. Hormone-naïve patients with biopsy-proven PC have been treated at Mayo Clinic by radical retropubic prostatectomy between January 1995 and December 1998 without neoadjuvant therapy. The study was approved by the Mayo Clinic Institutional Review Board. FFPE tissues were collected and total RNAs were isolated using a RecoverAll Total Nucleic Acid Isolation Kit (Thermo Fisher Scientific).

### Cell morphology analysis and photography

LNCaP and C4-2 cells were treated with enzalutamide for 28 days and the morphological images were acquired using the Leica DMI3000 B microscope (Wetzlar, Germany) from at least 3 random fields. Scale bars, 100 μm.

### Cell viability (MTS) assay

The MTS assays were performed according to manufacturer's instructions (Promega). Briefly, cells were plated in 96-well plates at a density of 2,000 cells per well. At the indicated times, 20 μl of CellTiter 96R AQueous Solution Reagent (Promega) was added to each well, and incubated for 2 h at 37°C in incubator and then was measured in a microplate reader at 490 nm.

### Chromatin immunoprecipitation (ChIP), ChIP-seq and data analysis

ChIP was performed as described previously [51] using anti-AR antibody (N20, Santa Cruz Biotechnology). LNCaP and C4-2 cells were treated with mibolerone, a synthetic androgen (1 nM) or ethanol for 3 days. ChIP-seq libraries were prepared using the methods as described previously [52] and high throughput sequencing was performed using the Illumina HiSeq2000 platforms at the Mayo Genome Core Facility. The data were analyzed using the following pipeline: (1) Mapping. ChIP-seq raw reads were aligned to reference genome (GRCh37/hg19) using Burrows-Wheeler Alignment (BWA) tool [53]. (2) Quality control. Subsequent to alignments, the following factors were evaluated to ensure that the sequencing data were of sufficient quality for downstream applications: the number of reads that can be mapped to unique locations in the genome, the number of nonredundant reads and the saturation test of sequencing depth. The saturation test was used to decide if current sequencing depth is sufficient to capture all protein binding locations. (3) Peak detection and visualization. MACS [54] was used to perform peak calling because it has been demonstrated to work very well with sharp peaks for transcription regulatory proteins (such as AR). BigBed and BigWig files were generated to facilitate both easy processing and high performance visualization with the UCSC genome browser or IGV. Integration of AR ChIP-seq data with other published epigenetic datasets such as histone ChIP-seq was performed using Epidaurus [55]. For ChIP-qPCR experiments, DNAs pulled down by antibodies were amplified by real-time PCR. Raw and processed data have been deposited into NCBI Gene Expression Omnibus with accession number GSE55032. Primer sequences are described in Supplementary Table 1.

### RNA-seq and data analysis

LNCaP and C4-2 cells were plated in medium described above and after 24 h, 10 μM enzalutamide or DMSO was added, and the cells were treated for 28 days. Total RNAs were isolated from cells using the methods as described previously [56]. Briefly, RNA was isolated using RNeasy Plus Mini Kit (Qiagen). High quality (Agilent Bioanalyzer RIN >7.0) total RNAs were employed for the preparation of sequencing libraries using Illumina TruSeq Stranded Total RNA/Ribo-Zero Sample Prep Kit. A total of 500-1,000 ng of riboRNA-depleted total RNA was fragmented by RNase III treatment at 37°C for 10-18 min and RNase III was inactivated at 65°C for 10 min. Size selection (50 to 150 bp fragments) was performed using the FlashPAGE denaturing PAGE-fractionator (Thermo Fisher Scientific) prior to ethanol precipitation overnight. The resulting RNA was directionally ligated, reverse-transcribed and RNase H treated.

Samples with biological duplicates were sequenced using the Illumina HiSeq2000 platform at the Mayo Genome Core Facility. Pre-analysis quality control was performed using FastQC (http://www.bioinformatics.babraham.ac.uk/projects/fastqc/) and RSeQC software [57] to ensure that raw data are in excellent condition and suitable for downstream analyses. Pair-end raw reads were aligned to the human reference genome (GRch37/hg19) using Tophat [58]. Genome-wide coverage signals were represented in BigWig format to facilitate convenient visualization using the UCSC genome browser. Gene expression was measured using RPKM (Reads Per Kilo-base exon per Million mapped reads) as described previously [59]. Correlation analyses between eRNA and mRNA expression were performed using Python and R scripts. EdgeR [60] was used to identify genes that were differentially expressed between CRPC and primary prostate tumors. Raw and processed data have been deposited into NCBI Gene Expression Omnibus with accession number GSE55032.

### Real-time RT-PCR

Total RNA was isolated with Trizol reagent (Thermo Fisher Scientific) and cDNA was prepared using SuperScript II reverse transcriptase (Thermo Fisher Scientific). Quantitative real-time PCR was performed with iQ SYBR Green Supermix on iCycleriQTM detection system (Bio-Rad) according to manufacturer's instructions. The relative expression level of RNA was calculated by normalizing to glyceraldehyde-3-phosphate dehydrogenase (*GAPDH*) levels using the 2^−ΔΔCT^ method. Primer sequences are described in Supplementary Table 1.

### 3C assay

The 3C assay was performed as previously described with some modifications [61]. Long- or short-term enzalutamide-treated LNCaP and C4-2 cells were fixed with 1% formaldehyde. Cell pellets were lysed and resuspended in restriction buffer for BanI and XhoI respectively for the loci of *NCAM2* and *MARC1* and treated with 0.3% SDS for 1 h at 37°C. Triton X-100 was added to a final concentration of 2% followed by overnight digestion of BanI and XhoI (1,500 U per 10^7^ cells) at 37°C. DNA ligation was performed for 4 h at 16°C. The ligated samples were reverse cross-linked with proteinase-K at 65°C overnight, followed by phenol/chloroform extraction and ethanol precipitation. The primers for PCR are provided in Supplementary Table 1.

### Cell infection by shRNA and western blot

Short hairpin RNAs (shRNA) specific for *NCAM2*, *FTO*, *MARC1*, *LUZP2* and non-specific (NS) shRNA were purchased from Open Biosystems. 293T cells were transfected with shRNA constructs using Lipofectamine 2000 (Thermo Fisher Scientific). Supernatant containing virus particles was harvested 2 days after transfection and used for infection of enzalutamide-resistant C4-2 cells. Cells were harvested 48 h after infection. For western blot, briefly, protein samples were denatured and separated by SDS-polyacrylamide gel electrophoresis (SDS/PAGE), and then transferred to nitrocellulose membranes (Bio-Rad). The membranes were immunoblotted with primary antibodies, horseradish peroxidase-conjugated secondary antibodies, and exposed to SuperSignal West Pico Stable Peroxide Solution (Thermo Fisher Scientific).

### Prostate cancer xenografts LuCaP35 and 35V

Patient-derived androgen dependent (AD) LuCaP35 and castration-resistant (or androgen independent, AI) LuCaP35V xenograft models were kindly provided by Dr. Robert L. Vessella (Department of Urology, University of Washington Medical Center, Seattle, WA). AD LuCaP xenografts were propagated in BALB/c nu/nu mice and AI xenografts were propagated in SCID mice. These experiments were performed in the laboratory of Dr. Donald Tindall at the Mayo Clinic. Mice were housed in the pathogen-free rodent facility at the Mayo Clinic. All procedures were approved by the Mayo Clinic Institutional Animal Care and Use Committee (IACUC).

### Statistics

Experiments were carried out with two or more replicates unless otherwise stated. Data are expressed as the mean ± standard deviation (SD) if not otherwise indicated, and compared using the independent Student's t-test. Values with p < 0.05 are considered statistically different.

## SUPPLEMENTARY TABLE


