# Supplementary material for: Alterations of androgen receptor-regulated enhancer RNAs (eRNAs) contribute to enzalutamide resistance in castration-resistant prostate cancer
Source: Oncotarget. 2016 May 21;7(25):38551–65. doi: 10.18632/oncotarget.9535 (PMC5122410; doi:10.18632/oncotarget.9535)
Supplement: Supplementary file 1 [file oncotarget-07-38551-s001.pdf]

SUPPLEMENTARY TABLE

Supplementary Table S1: Information of the primers used

**RT-qPCR eRNA primers**	
NCAM2 Forward	ATC CTT CCG GTT GCA GAC AC
NCAM2 Reverse	AGT GGC ACT CGC CAG TAT TA
FTO Forward	AGG GAA CCA GAA CAC AAC CAA T
FTO Reverse	AAA CCC CAG CCA ATG AAA ATG G
MARC1 Forward	GAG CCA CGG AAG GTT TGT GA
MARC1 Reverse	TCC TCA TTC CGA CTG TCA CG
LUZP2 Forward	TCT GGA AAG CAC AGC ACG TC
LUZP2 Reverse	GAT TGT TCT CTT GCG GTG CAG
**RT-qPCR mRNA primers**	
NCAM2 Forward	TGA GCC TCC TCC TCT CCT TC
NCAM2 Reverse	CAC CAA TCG CTG TAC ATG TG
FTO Forward	TAG CTG TGA AGG CCC TGA AGA
FTO Reverse	GTT GTG AAC CGG CCA AAA CA
*MARC*1 Forward	AGC AGA CTT GTT CCG ACC CA
MARC1 Reverse	TCC CAA GAA TCC TCT GCA TAG AC
LUZP2 Forward	AGC ACC AGA CAG GAC TAT GAA GA
LUZP2 Reverse	TTT TGG CAG ACT GCT CAT CGT TT
**3C Assay Primers**	
MARC1 Promoter Forward 1	GTG CCT GCA GGA CCA AGT GAC
MARC1 Control Forward 2	TCG GCA GAC TGC GGA AAG G
MARC1 Enhancer Forward 3	GCT GGG CAG GCT GTT CAC TG
MARC1 Promoter Reverse 1	CAG AGT TCT CCC GGC GGC
LUZP2 Promoter Forward 1	GCC CAA GGG GCT AAT ATT CAC T
LUZP2 Control Forward 2	TCT CAA AGG CAT GAC TTT CAG TCC
LUZP2 Enhancer Forward 3	GTG GGA AAG CCA AAT ATG TGC TCT C
LUZP2 Promoter Reverse 1	CGG GGG CAA GCT CTT TAC AG
NCAM2 Promoter Forward 1	CTA CTC CGG AAC TCA TAC CCT TTG C
NCAM2 Control Forward 2	CCC TCA CTG TTC TAC TAT GCT G
NCAM2 Enhancer Forward 3	GTG AGC CAC TGC GCC CAG
NCAM2 Promoter Reverse 1	AAT TCT CAT TCA GTG ACA CAT CTC TG
**AR ChIP primers**	
NCAM2 Forward	TC CTT CCG GTT GCA GAC AC
NCAM2 Reverse	GT GGC ACT CGC CAG TAT T
FTO Forward	GG GAA CCA GAA CAC AAC CA
FTO Reverse	CCC CAG CCA ATG AAA ATG G
MARC1 Forward	GAG CCA CGG AAG GTT TGT G
MARC1 Reverse	CC TCA TTC CGA CTG TCA CG
LUZP2 Forward	CT GGA AAG CAC AGC ACG TC
LUZP2 Reverse	T TGT TCT CTT GCG GTG CAG
